# Impact of phenolic composition on hepatoprotective and antioxidant effects of four desert medicinal plants

**DOI:** 10.1186/s12906-015-0919-6

**Published:** 2015-11-09

**Authors:** Naglaa Gamil Shehab, Eman Abu-Gharbieh, Fatehia A. Bayoumi

**Affiliations:** Department of Pharmacognosy, Faculty of Pharmacy, Cairo University, Cairo, Egypt; Department of Pharmacology and Toxicology, Dubai Pharmacy College, Al-Nahda 3, Dubai, UAE; Department of Pathology, Dubai Medical College, Al-Nahda 3, Dubai, UAE

**Keywords:** Antioxidant, Flavonoids, Hepatoprotective, Phenolic acids

## Abstract

**Background:**

Flavonoids and other polyphenols play a protective role in liver diseases and possess a high antioxidant capacity.

**Objective:**

To compare and evaluate the antioxidant and hepatotoprotective activities of 4 deserts plants, *Fagonia indica* Burm. f., *Calotropis procera* R.Br., * Zygophylum hamiense* Schweinf. and *Salsola imbricata* Forssk. in correlation to their composition especially their phenolic content.

**Methods:**

The influence of extracting solvent on total phenolic and flavonoidal contents was assessed spectrophotometrically. The flavonoid and other polyphenolic components of the methanol extracts were analyzed by RP-HPLC. DPPH radical scavenging potential of the different extracts was estimated. The hepatoprotective and antioxidant activities of the extracts against CCl_4_-induced hepatotoxicity in mice were evaluated.

**Results:**

The flavonol quercitrin and rosmarinic acid were major in the *F. indica, C. procera* and *S. imbricata *samples, while rutin prevailed in that of *Z. hamiense*. The ethanolic and methanolic extracts showed noticeable DPPH radical-scavenging activity as compared to ascorbic acid. Assessment of liver enzymes revealed that oral administration of the extracts did not show any evidence of hepatotoxicity. Moreover, protection against CCl_4_-induced liver damage was evident upon administration of three plants extracts namely, *F. indica, C. procera* and *S. imbricata.*

**Conclusion:**

Overall, hepatotoxicity induced by CCl_4_ was effectively prevented by the three plants extracts through scavenging of free radicals and by boosting the antioxidant capacity of the liver. The protective effect of the plants could be attributed to their high quercitrin and rosmarinic acid contents.

## Background

Human beings are daily exposed to various compounds that can cause serious diseases either *per se* or through their metabolic activation to highly reactive substances such as reactive oxygen species (ROS). Free radical induced lipid peroxidation is regarded as one of the main causes of cell membrane damage leading to various pathological conditions [1, 2]. Liver disorders are considered among the major world health problems [3]. Despite their prevalence, morbidity and mortality rates, their current medical management is still considered inadequate. Until now, no therapy shows complete success in preventing the disease progression [4]. Besides, the newly developed drugs used in management of chronic liver diseases are usually associated with various, and sometime intolerable, side effects [5]. Consequently, medicinal plants, especially those with traditional use, have always been considered as a rich source of new effective drugs which could help in ameliorating liver conditions.

Among plant metabolites, phenolics are reputed to play a noticeable protective role against several health disorders [6]. Phenolics possess various biological activities, for instance, antiulcer, anti-inflammatory [7], antidiabetic [8], antioxidant, cytotoxic and antitumor [9, 10].

*Fagonia indica* Burm. f. (Mushikka or white spine) (Zygophyllaceae) is a widely distributed plant in the deserts of Asia and Africa. It has been reported as medicinal herb in the scientific literature. In an earlier study, the main author reported that the plant could be considered as safe and that it contained a variety of bioactive flavonoids, sterols and triterpenoids; its alcoholic extract was found to exhibit antitumor, antimicrobial and analgesic activities [11]. Furthermore, the methanolic extract of an Indian sample of the plant was proven to exert a hepatoprotective effect in rats; however, the mechanism of action has not yet been explored [12].

*Calotropis procera* R. Br. (Asclepiadaceae), known as Giant milkweed and locally called Al-ashkhar [13], has been used for treating various diseases like rheumatism, filariasis and skin disorders [14] and its leaf to treat jaundice [15]. The flowers extract have been used for treating spleen, liver and abdomen diseases [16]. Additionally, various extracts of its different parts showed antibacterial and *in-vitro* and* in-vivo* antioxidant activities [17–20]. Earlier phytochemical investigation of *C. procera* revealed the presence of cardenolides, flavonoids, steroids and saponins [21, 22]. The composition of the volatiles, lipoids and flavonoids of its flowers were previously investigated by the author [23].

*Zygophyllum* species (Family Zygophyllaceae) are used as anthelmintic and for management of diabetes mellitus [24, 25]. The aqueous extract of *Zygophyllum album* showed in *vivo* antihyperglycemic, antioxidant and antihyperlipidemic effects [26] as well *in-vitro* and *in-vivo* antioxidant properties and phenolic contents of *Zygophyllum* species were investigated [27–29]. *Zygophyllum hamiense* Schweinf. spreads largely along the Arabian Gulf area and grows on salt accumulated land. The dead trees are commonly used as firewood and the sprouts as camel food [30]. Yet, there are no available reports regarding either the composition or biological activities of the *Zygophyllum hamiense* Schweinf.

*Genus Salsola* (Family Chenopodiaceae or Amaranthaceae) exhibited significant *in-vitro* antioxidant activities [31–33]. Flavonoid and other phenolic compounds from different species of *Salsola* have been reported. In addition, triterpenes with significant antioxidant activity were isolated [34, 35]. *Salsola imbricata* Forssk. (Arabic names: Harm), is a shrub wild growing in Middle East deserts; it is distributed throughout Central and Southwest Asia, North Africa, and Mediterranean countries [36, 37]. Previous phytochemical investigations and biological study of the plant were limited. Two triterpenoidal saponin glycosides were isolated and identified from the roots of the Egyptian plant [38]. The phenolic profile of the alcoholic extract of the plant was analyzed and its contraceptive effect in male albino rats previously evaluated by the authors [39].

The selected plants are growing and existing in the deserts. The deserts plants almost contain variety of secondary metabolites like flavonoids and phenolic acids to protect themselves from herbivores. Thus it was valuable and interesting to perform a comparative study on some selected desert plants from different genus, *Fagonia indica* Burm. f*.*, *Calotropis procera* R.Br., *Zygophylum hamiense* Schweinf. and *Salsola imbricata* Forssk., and to correlate their biological activities such antioxidant and hepatotoprotective to their phenolic composition.

## Methods

### Chemicals and drugs

Methanol, ethanol, acetone and ethyl acetate were purchased from Fisher Scientifics (UK) & Scharlam. Carbon tetrachloride (CCl_4_), 2,2-diphenyl-1-picrylhydrazyl (DPPH), sodium carboxymethylcellulose (CMC), Biochemical kits for determination of glutathione peroxidase (GPx), superoxide dismutase (SOD), Catalase (CAT), Thiobarbituric acid reactive substances (TBARS) were purchased from Sigma Chemical Co. (St. Louis, MO, USA) and Folin-Ciocalteu reagent was obtained from Merck (Darmstadt, Germany). All other chemicals were of analytical grade.

### Plants material

Whole plants of *Fagonia indica* Burm. f*., Zygophylum hamiense* Schweinf., *Salsola imbricata* Forssk*.* and leaves of *Calotropis procera* R. Br. were collected during September 2012 from Muhaisnah desert, Dubai, UAE. The samples were kindly identified and authenticated by Prof. Hassnaa Ahmed Hosny, Department of Botany, Faculty of Science, Cairo University, Egypt. Voucher specimens were kept at the Herbarium of the Pharmacognosy Department, Faculty of Pharmacy, Cairo University. Samples, air-dried in shade, were powdered and preserved for further study.

### Experimental animals

#### Acute toxicity

The acute toxicity studies of both *F. indica* and *S. imbricata* have been previously reported [11, 39]. Male albino mice weighing 20–25 g (10 per group) were used to estimate the acute toxicity of the other two plants *viz*., *Z. hamiense* and *C. procera*. LD_50_ was estimated using 50 % death within 72 h following oral administration of the extracts at different doses (250, 500, 1000, 2500 and 5000 mg/kg). The number of animals, which died during this interval, was expressed as a percentile, and the LD_50_ determined by probit test using a death percent versus doses’ log [40].

#### Treatment protocol

Eighty four healthy male albino mice of weights ranging from 30-35 g were used. Animals were kept under the same standard hygienic conditions (temperature 22.0 ± 2.0 °C, relative humidity 50–60 %, with 12 h day/night lighting cycle), fed with well-balanced normal diet and water supplied *ad libitum*. They were left for a period of one week for accommodation before performing the experiments. All animals’ investigations were performed in accordance with the ethical standards for the proper care and use of laboratory animals and upon approval of the Research Ethical Committee of the Dubai Pharmacy College, Dubai, United Arab Emirates.

### Plants extracts

The four air-dried powdered plants materials (500 g, each) were exhaustively extracted by cold maceration in 70 % ethanol (3 L X 2). The solvents were evaporated under reduced pressure at 50 °C. The residual weights for *F. indica, C. procera,, Z. hamiense* and *S. imbricata* amounted to 80.0, 44.5, 28.0 and 20.0 g, respectively. These dried extractives were saved and used for biological evaluation.

### Standardization of the plants extracts

#### Colorimetric monitoring of phenolic content in different extracting solvents

Solvents of different polarities, namely: 70 % ethanol, methanol, acetone and ethyl acetate were individually used for extraction of the air-dried powdered plant materials (100 g, each).

The efficiency of the extracting solvent was monitored by colorimetric estimation of total phenolic and flavonoid contents using a spectrophotometer (UV-1700 Pharma Spec, Shimadzu, Japan). All experiments were carried out in triplicate.

The *total phenolic contents* were determined by using Folin-Ciocalteu reagent as described by Singleton and Rossi [41] and modified by Oktay et al. [42]. Results were expressed as mg/g gallic acid equivalent, calculated on dry weight of plant material; serial dilutions of gallic acid (10, 20, 30, 40, and 50 $\mu$g/mL) were used for establishment of the calibration curve. Aliquots (1 mL, each) of tested samples and standard were, separately, added to a volumetric flask containing 9 mL of water followed by addition of 1 mL of Folin-Ciocalteu reagent and the reaction mixture was carefully blended by vortex. After 5 min, 10 mL of 7 % sodium carbonate was added to the mixture which was further incubated for 90 min, at room temperature. Finally, the absorbance was determined at 750 nm against the reagent blank.

The *total flavonoid content* of the prepared extracts was measured, spectrophotometrically, by the aluminum chloride method, quercetin being used as standard, by adopting the procedure described by Dewanto et al. [43]. The plants extracts (0.1 mL each) were added to 0.3 mL distilled water followed by 5 % NaNO_2_ (0.03 mL) and the reaction mixture was left for 5 min, at 25 °C. Aluminium chloride (0.03 mL, 10 %) was then added and the mixture left for another 5 min, then treated with 0.2 mL of 1 mM NaOH, and finally diluted to 1 mL with water and the absorbance of the yellow colour produced read at 510 nm.

#### HPLC analysis of phenolics

The phenolic composition of the methanolic extract of *S. imbricata* was previously analysed by the authors [39]. Methanolic extracts of the other three plants (*F. indica*, *C. procera* and *Z. hamiense*) were investigated in aliquots of 1g each *via* RP-HPLC on a Hewlett Packard HPLC System (HP 1050HPLCDADw/Data System). Analyses were carried out at operating conditions suitable for detection of either phenolic acids or flavonoids [44, 45]. For determination of phenolic acids, the apparatus was equipped with an Alltima C18 column (particle size 5 mm, 150 × 4.6 mm) and Alltima C18 guard column (5 mm) (Alltech, USA), the UV detector being set at 280 nm. Meanwhile, the separation of flavonoids was carried out on a Hypersil-ODS C18 column (particle size 5 $\mu$m, 4.6 × 250 mm) and the UV detector was set at 330 nm. All analyses were performed at 35^∘^C; gradient elution was employed using acetonitrile-acetic acid mixtures as mobile phase, at a flow rate of 1 mL/min, and the injected volume was 10 $\mu$L for both standard and tested samples. Authentic reference samples were prepared by diluting stock solutions with methanol to afford a 50 $\mu$g/mL final concentration. Identification of individual components was performed by comparing their retention times with those of the available standards similarly analyzed. Quantification was based on peak area computation using the external standard method. All analyses were carried out in triplicate. Samples were analyzed at 280 and 330 nm, respectively.

### Antioxidant activity

#### 2,2-Diphenyl-1-picrylhydrazyl (DPPH) radical scavenging assay

The free radical-scavenging activities of the extracts of the four plants (prepared in the following solvents: 70 % ethanol, methanol, acetone, and ethyl acetate) were measured through the hydrogen donating or radical-scavenging ability using the stable DPPH radical. The assay was performed in a 96-well microtiter plate using the modified previously described method [46]. Hundred μl of each of the samples and the standard solutions were mixed with 100 μl of 0.1 mM ethanolic DPPH solution in the wells. The reaction mixtures were shaken vigorously and incubated in dark for 30 min at 37 °C. The absorbance was measured at 517 nm using UV–vis microplate reader. The percentage inhibition (%) of the DPPH radical by the samples was calculated using the following formula:

$$ \%\ \mathrm{inhibition} = \left[{\mathrm{A}}_0 - \left({\mathrm{A}}_1 - {\mathrm{A}}_2\right)\right]/{\mathrm{A}}_0\times 100\% $$

Where A_0_ is the absorbance of the control, A_1_ is the absorbance in the presence of the sample and A_2_ is the absorbance of the sample under identical conditions as A_1_ with ethanol instead of DPPH solution. Ascorbic acid (AA) was used as a reference compound. IC_50_ values were calculated. Samples were analyzed in triplicate.

### Experimental design

The residues of the ethanolic extracts for the four plants were suspended in 1 % CMC. The animals were randomly assigned to 14 groups, of 6 animals each (*n* = 6). Table 1 describes the animal grouping with their corresponding treatment.

**Table 1:** Animals groups and corresponding treatment with vehicle, tested samples and carbon tetrachloride

	Oral treatment	Frequency	i.p. CCl_4_ injection
Control	Vehicle	daily	-
CCl_4_ control	Vehicle	daily	1.0ml/kg
*F. indica* extract	10 mg/kg	Twice daily	-
	5mg/kg	daily	1.0 ml/kg
	10 mg/kg	daily	1.0 ml/kg
*Z. hamiense* extract	500 mg/kg	Twice daily	-
	250 mg/kg	daily	1.0 ml/kg
	500 mg/kg	daily	1.0 ml/kg
*C. procera* extract	200 mg/kg	Twice daily	-
	100 mg/kg	daily	1.0 ml/kg
	200 mg/kg	daily	1.0 ml/kg
*S. imbricata* extract	500 mg/kg	Twice daily	-
	250 mg/kg	Daily	1.0 ml/kg
	500 mg/kg	Daily	1.0 ml/kg

The first group served as normal control and during the experiment received vehicle only (1 % CMC). The second group was given 1 % CMC solution for 14 days before CCl_4_ intoxication and served as a hepatotoxicity control group. For each plant, three groups were devoted; the first was treated with the plant extract twice daily for 14 days while the second and the third groups were given the plant extract in two different doses for 14 days as shown in Table 1. After the 14-days treatment period, hepatic injury was induced by intraperitoneal injection of 1.0 ml/kg of CCl_4_ and the mice were sacrificed six hours after the last treatment.

### Assay of liver enzyme

Blood samples were collected from the hearts with the use of 5 ml sterile syringe individually for each mouse and transferred into non-heparinized tubes immediately and used later for the analyses of liver enzymes: alanine aminotransferase (ALT), aspartate aminotransferase (AST) and alkaline phosphatase (ALP).

### Estimation of oxidative parameters

Liver samples were surgically removed from the mice immediately and stored in -80° for further antioxidant enzyme assay including activity of catalase, superoxide dismutase, glutathione peroxidase and TBARS as per the method described by Abu-Gharbieh et al. [47]. The levels of total protein were determined in the serum of experimental animals by using the Lowry method and the bromocresol green method, respectively [48, 49].

### Histopathological study

Liver samples were suspended in 10 % formaldehyde for histological evaluation. These tissues were processed and embedded in paraffin wax. Sections of 5 μm in thickness were cut and stained with hematoxylin and eosin (H&E) and Periodic acid-Schiff (PAS) stains.

### Statically analysis

The results were reported as Mean ± Standard Deviation (S.D) from three repeated determination. The data obtained were statistically analyzed using One-way analysis of variance ANOVA, followed by Dunnett’s multiple comparison test (DMCT). *P*-value of < 0.05 was considered as statistically significant.

## Results

### Standardization of the plants extracts

#### Influence of extracting solvent on total phenolic and flavonoid contents

Different solvents were used to select the most efficient, safe and applicable solvent for phenolic compounds extraction as shown in Table 2.

**Table 2:** Total Flavonoid and phenolic acid contents of the different extracts of *Fagonia indica*, *Calotropis procera*, *Zygophyllum hamiense* and *S.imbricata*

Plant name	Total flavonoid content g quercetin/100 g				Total phenolic content mg GAE/g			
	Methanol extract	Ethanol extract	Acetone extract	Ethyl acetate extract	Methanol extract	Ethanol extract	Acetone extract	Ethyl acetate extract
*F. indica*	0.22	3.00	0.32	0.10	3.91	4.00	2.1	0.75
*C. procera*	0.12	0.3	0.90	0.10	3.13	4.00	4.00	0.92
*Z. hamiense*	0.38	0.14	1.48	0.11	2.60	3.61	1.20	4.00
*S. imbricata* ^a^	0.571	0.217	0.374	0.11	2.60	0.64	4.00	0.93

^a^Results previously reported [39]

Spectrophotometric evaluation of the total phenolic content (expressed as mg gallic acid equivalent (mg GAE)/g dry plant material) and flavonoid content (as quercetin g/100 g dry plant material) in the extracts of *F. indica*, *C. procera* and *Z. hamiense* revealed variable efficiency. Ethanol was found to be the best solvent for extracting the *F. indica* sample with highest concentration of phenolics (4 mg GAE/g dry plant wt.) and flavonoids (3 g quercetin (Q) % w/dry plant wt.). Concerning *C. procera*, the highest flavonoid content was detected when using acetone (0.9 g Q % w/dry wt.), while both ethanol and acetone extracts were found the richest in total phenolics (4 mg GAE/g dry wt.). On the other hand, the maximum flavonoid amount (1.48 %) was extracted with acetone in *Z. hamiense*, meanwhile ethyl acetate and ethanol appeared of close efficiencies for solubilisation of total phenolics (4 and 3.61 mg GAE/g dry plant wt., respectively)*.* Finally, among the tested plants and including *S. imbricata*, the most enriched sample in both total phenolics and flavonoids was *F. indica* (4 mg GAE/g of plant dry wt. and 3 g % w/dry wt., expressed as quercetin respectively).

#### RP-HPLC profiling of phenolics

RP-HPLC analysis and total phenolic and flavonoid contents of *S. imbricata* were previously determined [39]. RP-HPLC analyses of the methanolic extracts of the remaining three plants, *F. indica, C. procera,* and *Z. hamiense,* allowed the identification and quantitation of several phenolics. Total of 14 components were identified at 280 nm in both *C. procera* and *F. indica* (corresponding to 10.297 and 7.955 % of the total composition, respectively) and 13 components in *Z. hamiense* (corresponding to 19.52 %) as shown in Table 3. Among these, the identified phenolic acids were 10 in *F. indica* (representing 4.84 %), 9 in *C. procera* (6.274 %) and 8 in *Z. hamiense* (11.35 %). Ellagic and non-phenolic benzoic acids (1.2 and 1.02 %) were the prevalent in *F. indica* while in *C. procera,* benzoic and salicylic acids (1.59 and 1.53 %) were the major. On the other hand, chlorogenic and gallic acids were predominant in *Z. hamiense* (2.31 and 2.02 %). In contrast, 9 phenolic acids (9.734 %) with prevalence of coumaric acid (4.251 %) were detected in *S. imbricata* [39]*.* On the other hand, by setting the detector at $\lambda$ = 330 nm, 8 components were identified in both *F. indica* and *C. procera* among which 7 were of flavonoidal nature with major quercitrin (5.29 and 4.16 % respectively) while 5 components only were determined in *Z. hamiense* among which 4 were of flavonoidal nature with major rutin (10.71 %) while quercitrin was the minor (0.77 %) as shown in Table 4. Alternatively, 7 flavonoidal components with major quercitrin (12.692 %) were previously detected in *S. imbricata*. Besides, rosmarinic acid was detected in all samples under investigation with relatively appreciable amounts (Table 4).

**Table 3:** Phenolics identified by RP-HPLC analysis (at $\lambda$ = 280 nm) of the methanolic extracts of *Fagonia indica, Calotropis procera, Zygophyllum hamiense* and *S.imbricata*

Retention time	Identified constituent	Relative area %			
		*F. indica*	*C. procera*	*Z. hamiense*	*S. imbricata* ^a^
6.81	Pyrogallol	0.145	0.173	1.94	-
6.92	Gallic acid	0.05	0.27	2.02	0.145
8.235	Protocatechuic acid	0.12	0.504	0.97	0.068
8.444	Catechin	-	0.38	1.81	0.461
8.593	Chlorogenic acid	0.46	0.12	2.31	0.377
8.950	Catechol	0.16	0.44	2.57	0.329
10.040	Caffeic acid	0.25	-	1.54	1.759
11.073	Vanillic acid	0.37	0.82	1.37	0.286
11.620	Ferulic acid	0.34	0.36	0.84	1.323
12.466	*Salicylic acid*	0.73	1.53	1.1	1.154
12.943	*Ellagic acid*	1.20	1.20	-	-
13.127	Benzoic acid	1.02	1.59	1.51	2.306
13.789	Coumaric acid	0.51	0.52	1.2	4.251
14.980	Cinnamic acid	0.81	0.95	-	0.371
18.657	Chrysin	1.79	1.44	0.34	1.074
Total identified constituents		7.955	10.297	19.52	13.904

^a^Results previously reported [39]

**Table 4:** Phenolics identified by RP-HPLC analysis (at $\lambda$ = 330 nm) of the methanolic extracts of *Fagonia indica*, *Calotropis procera*, *Zygophyllum hamiense* and *S.imbricata*

Retention time	Identified constituent	Relative area %			
		*F. indica*	*C. procera*	*Z. hamiense*	*S. imricata* ^a^
3.83	Quercetin	0.034	0.08	0.04	0.031
11.78	Rosmarinic acid	2.83	2.68	2.33	2.734
12.06	Hesperidin	1.21	2.22	-	1.854
12.44	Rutin	-	1.72	10.71	2.101
13.267	Quercitrin	5.29	4.16	0.77	12.692
14.576	Naringenin	1.36	1.15	0.94	1.300
14.952	Hesperitin	1.41	-	-	0.730
15.147	Kampferol	1.61	1.5	-	-
16.167	Apigenin	1.00	0.21	-	0.474
Total identified constituents		14.744	13.72	14.79	21.916

^a^Results previously reported [39]

### DPPH free radical scavenging activity

DPPH is a stable free radical and its noticeable purple color shows absorption at 517 nm. Antioxidants scavenge the free radical by donating a hydrogen atom and the color of the DPPH assay solution becomes yellowish, resulting in a decrease of the absorbance. DPPH free radical scavenging activity is considered as *in vitro* screening for possible *in vivo* antioxidant potentialities.

**Fig. 1:**
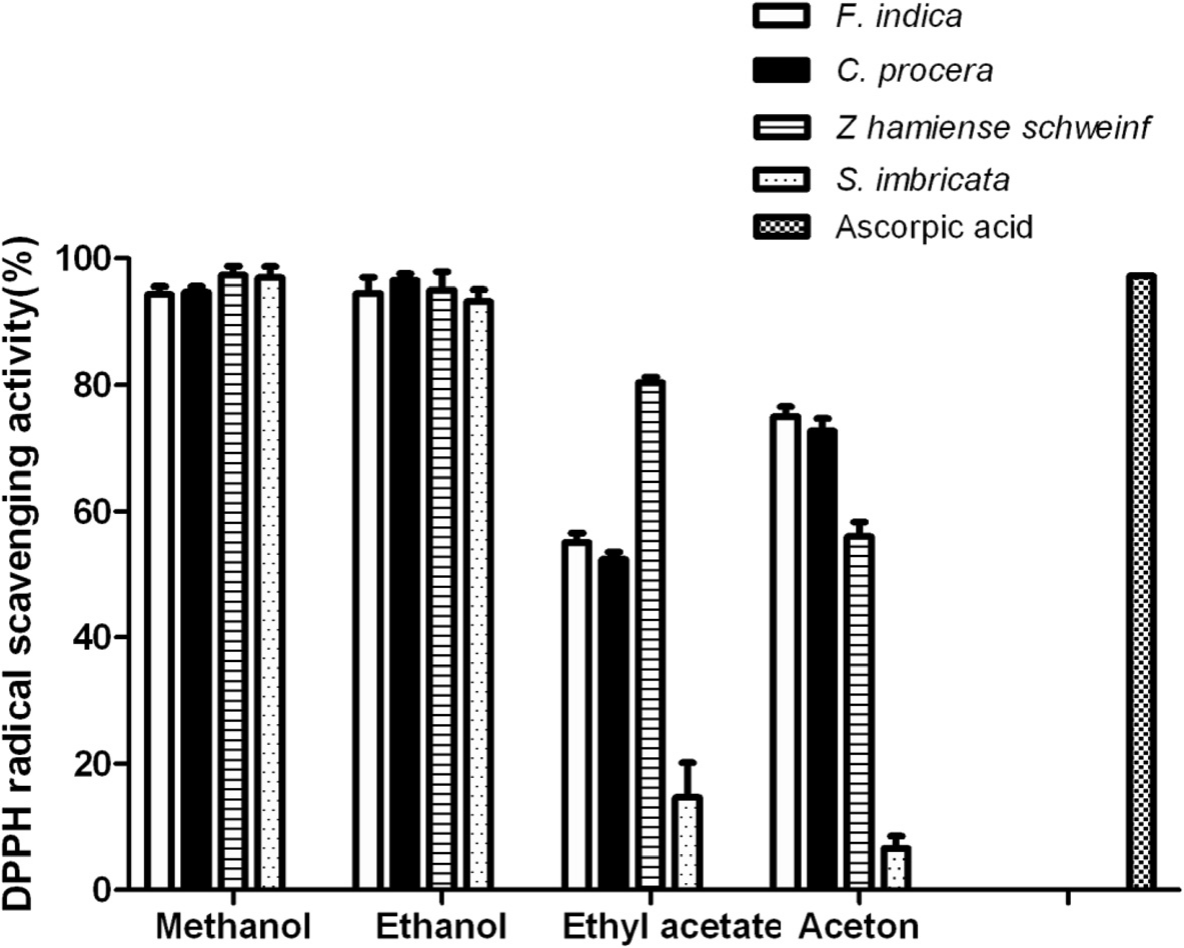
DPPH radical scavenging activity of several plants extracts

The four plants were tested and the results are presented in Fig. 1. All extracts were found to be potent DPPH free radical scavengers and the highest activity among all investigated plants samples was observed for the ethanolic and methanolic extracts as shown in Fig. 1.

### Acute toxicity study

The acute toxicity study was essential to evaluate the plants extracts safety and to determine the tested doses.

The LD_50_ of both *F. indica* and *S. imbricata* extracts had been previously reported and they were found to be safe up to 4 and 5 g/kg respectively [11, 39]. *Z. hamiense* was found to be safe up to dose 5 g/kg while *C. procera* leaves extract was safe up to 3 g/kg. No signs of morbidity or behavioral changes in any of the treated groups of animals during the period of observation. The safety margin of the ethanolic extracts of the plants under investigation is highly encouraging the biological evaluation.

### Effects on liver enzymes and histological findings

The plants extracts were given twice daily over two weeks in order to evaluate any possible hepatotoxicity caused by the plants extracts themselves. Moreover, this was useful to evaluate the effect of the extracts on the antioxidant enzyme system apart from the CCl_4_ challenge.

**Fig. 2:**
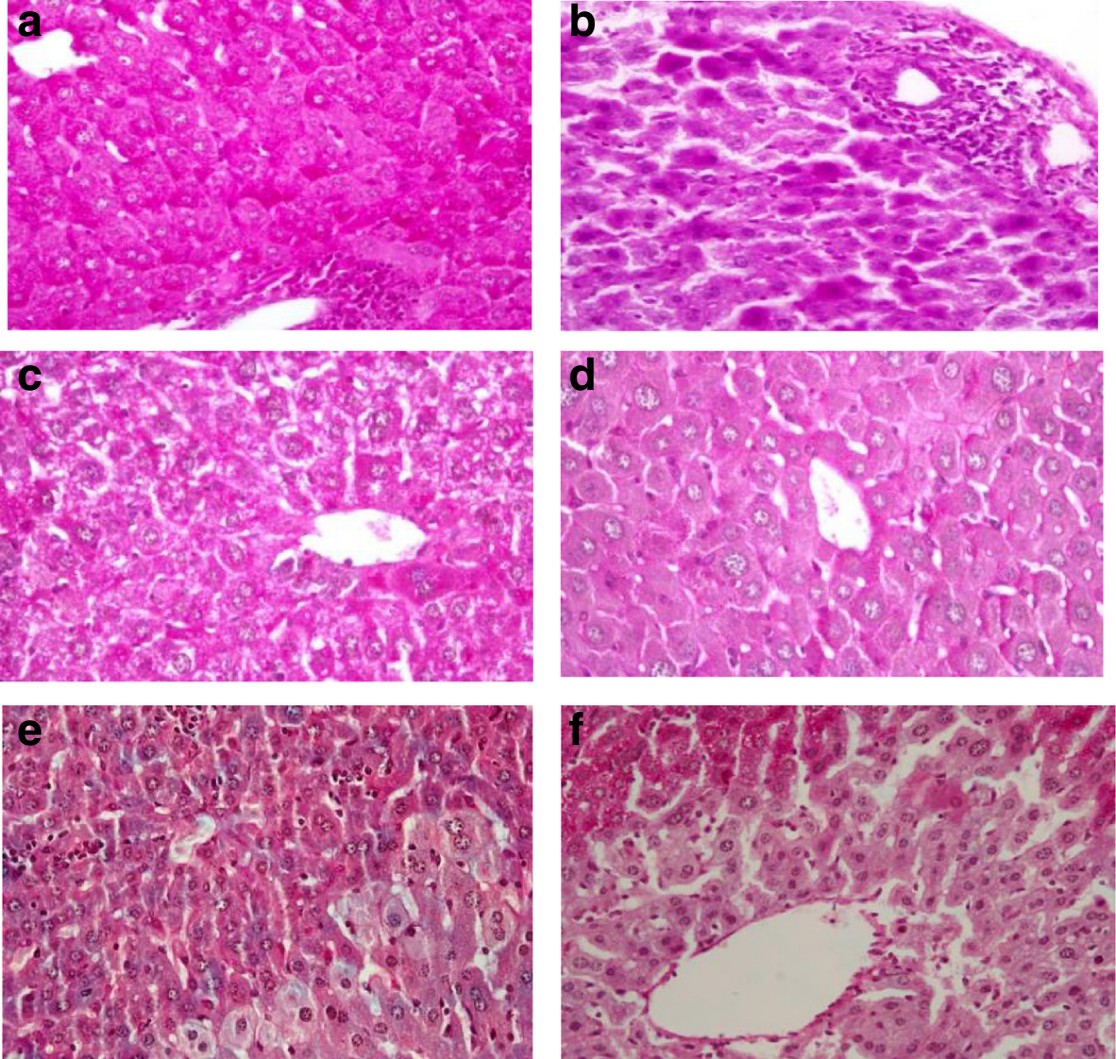
Histopathological findings of mice liver sections (PAS x400); (**a**) treated with 10 mg/kg *F. indica* twice daily showing excess glycogen synthesis; (**b**) negative control group treated with CCl_4_ alone showing dense periportal and lobular lymphocytic infiltrate with pyknotic nuclei within necrotic hepatocytes in periportal areas and some other cells show degenerative changes; (**c**) treated with 10mg/kg *F. indica* and CCl_4_ showing normal regenerating hepatocytes sometimes binucleated; (**d**) treated with 200mg/kg *C. procera* and CCl_4_ showing normal glycogen storage, stimulates regeneration and renewal of hepatocytes; (**e**) treated with 500mg/kg *S. imbricata* and CCl_4_ showing slight glycogen depletion and (**f**) treated with *Z. hamiense* Schweinf and CCl_4_ showing glycogen depletion in centrizonal area and more glycogen and ballooning degeneration on peripheral zone

Treating the animals twice daily with the plants extracts alone over two weeks, did not cause any significant elevation on both the ALT and AST as shown in Table 5. On the other hand, significant reduction in ALP levels was observed by administration of the ethanolic extracts of the four plants. This indicates that no possible cholestasis occurred at the dose levels tested since a rise in plasma ALP level is usually a characteristic feature in cholestatic liver disease [50]. Moreover, histological assessment revealed that the hepatocytes maintained its architecture with normal glycogen storage. This gave evidence that the four plants did not produce any harmful on the hepatocytes as shown in Fig. 2a. Beside this, treating the animals with the plants extracts did not show any significant enhancement of the antioxidant enzyme system.

**Table 5:** Evaluation of the hepatotoxic effect of the plant extracts on the biochemical parameters with twice daily oral administration in mice

	Control	*F. indica*	*Z hamiense*	*C. procera*	*S. imbricata*
ALT	290.1 ± 54.0	350.2 ± 113.6	155.3 ± 65.5	211.0 ± 35.8	253.3 ± 24.0
AST	850.0 ± 63.2	800.0 ± 235.8	520.3 ± 220.0	404.2 ± 37.2^b^	653.3 ± 78.8
ALP	180.0 ± 20.3	113.3 ± 16.7^a^	105.6 ± 15.4^a^	98.2 ± 9.3^b^	60.0 ± 23.1^b^
CAT kU/g protein	462.0 ± 21.1	461.6 ± 14.9	428.750 ± 10.3	413.950 ± 11.4	486.3 ± 18.0
GSH-Px U/g protein	1.3 ± 0.1	1.3 ± 0.05	1.3 ± 0.07	1.6 ± 0.06	1.31 ± 0.14
SOD μg/g protein	34.6 ± 2.9	35.5 ± 2.7	32.8 ± 1.1	35.5 ± 1.4	32.1 ± 1.4
TBARS (nmol/g prot.)	0.49 ± 0.02	0.39 ± .03	0.046 ± 0.03	0.038 ± 0.02	0.035 ± 0.4

^a^Significant difference (*p* < 0.05) compared to control group; ^b^Significant difference (*p* < 0.01) compared to control group

Results in Table 6, showed that CCl_4_ caused sharp and significant elevation in liver enzymes, ALT and AST by 598 % and 204 % respectively compared to control group (*p* < 0.01), while ALP was not significantly affected. Histological data revealed a dense periportal and lobular lymphocytic infiltrate with diffused pyknotic nuclei within necrotic hepatocytes in periportal areas. Fig. 2b.

**Table 6:** Evaluation of the protective effect of the plants extracts on the biochemical parameters of liver in CCl_4_-induced hepatic damage in mice

	Control	CCl_4_ control	*F. indica*		*Z hamiense*		*C. procera*		*S. imbricata*	
			5 mg/kg	10 mg/kg	250 mg/kg	500 mg/kg	100 mg/kg	200 mg/kg	250mg/kg	500mg/kg
ALT (U/L)	290.1 ± 54.0	1736.7 ± 161.2^a^	2166.7 ± 157.8^b^	1064.0 ± 264.9^c^	1416.7 ± 105.8	950.3 ± 84.4	1054.3 ± 117.5^c^	801.7 ± 76.0^c^	1135.0 ± 127.9^c^	476.7 ± 151.0^c^
AST(U/L)	850.0 ± 63.2	1736.7 ± 215.1^a^	1310.0 ± 181.5^b^	926.2 ± 258.6^c^	756.7 ± 128.6^c^	783.3 ± 80.1^c^	787.8 ± 69.6^c^	922.7 ± 65.4^c^	647.5 ± 62.8^c^	530.0 ± 120.8^c^
ALP(U/L)	180.0 ± 20.3	150.2 ± 55.7	125.0 ± 22.5	124.3 ± 17.4	106.7 ± 24.0	113.3 ± 29.1	51.8 ± 4.7	59.5 ± 6.1 ^c^	110.0 ± 14.7	63.3 ± 8.8
CAT kU/g protein	462.0 ± 21.1	241.3 ± 11.4^a^	307.2 ± 9.2^c^	392.200 ± 9.4^c^	225.1 ± 10.5	259.1 ± 5.1	324.7 ± 25.8^b^	335.0 ± 25.0^c^	356.2 ± 24.1^c^	374.9 ± 10.7^c^
GSH-Px U/g protein	1.3 ± 0.1	0.85 ± 0.06^a^	1.1 ± 0.2	1.2 ± 0.2	0.72 ± 0.1	0.94 ± 0.2	1.4 ± 0.06	1.4 ± 0.06	0.91 ± 0.06	1.39 ± 0.19^b^
SOD μg/g protein	34.6 ± 2.9	22.0 ± 2.4^a^	26.7 ± 1.8	32.9 ± 1.6^c^	21.1 ± 1.7	23.6 ± 1.5	22.7 ± 1.2	31.5 ± 1.1^b^	24.8 ± 0.5	26.4 ± 1.3
TBARS (nmol/g prot.)	0.049 ± 0.02	0.54 ± 0.20^a^	0.12 ± 0.09^c^	0.085 ± 0.05^c^	0.42 ± 0.2	0.36 ± 0.4	0.14 ± 0.07^c^	0.06 ± 0.01^c^	0.11 ± 0.05^c^	0.071 ± 0.02^c^

^a^Significant difference (*p* < 0.01) compared to control group; ^b^Significant difference (*p* < 0.05) compared to CCl4 treated group; ^c^Significant difference (*p* < 0.01) compared to CCl4 treated group

Pre-treating the mice with *F. indica, C. procera and S. imricata* extracts at the highest and lowest doses for 14 days prior CCl_4_ administration showed significant reduction in serum levels of ALT, AST but not ALP enzymes in a dose response manner (*p*-value less than 0.05 and 0.01, respectively). Those findings are supported by the histological features; hepatocytes renewal and regeneration with mild glycogen depletion were observed with *F. indica, C. procera* and *S. imbricata* only as shown in Figs. 2c, d and e. On the other hand, pretreating the animals with *Z. hamiense* extract at both doses showed significant reduction in AST level (*p* < 0.01) though ALT and ALP levels were not changed (*p* > 0.05). Furthermore, histological study showed centralized gross glycogen depletion (Fig. 2f).

### Effects on antioxidant enzymes and TBARS contents

Hepatotoxicity induced by CCL_4_ is characterized by suppression of the antioxidant defense system [51–53] and increased lipid peroxidation [51].

Administration of CCl_4_ markedly depleted the antioxidant enzymes (CAT, GSH-Px and SOD) in the mice livers (Table 6). Nevertheless, CCl_4_ increased significantly (*p* < 0.01) the hepatic lipid peroxidation that is expressed by high TBARS content. Whereas the administration of plants extracts twice daily for two weeks did not result in significant enhancement of the antioxidant enzymes nor reduction in the TBARS content as shown in Table 5.

Pretreating the animals with the plants extracts at different doses opposed significantly the reduction in the antioxidant enzymes and reduced markedly the TBARS content induced by CCl_4_ except for *Z. hamiense* extract that has no potential effects on the antioxidant enzymes as well as the TBARS content.

## Discussion

Liver diseases are one of the major causes of morbidity and mortality and affects people of all ages throughout the world especially in the Arab countries. The drugs that are currently available to treat this condition pose serious drawbacks [5], which justifies the search for new hepatoprotective agents. In this context, the use of plants extracts and isolates therefrom with hepatoprotective properties can provide beneficial means for prevention and treatment of liver conditions.

Alanine aminotransferase (ALT), aspartate aminotransferase (ASL) are present mostly in the hepatic and biliary cells [54]. These enzymes are usually released from the hepatocytes and leak into circulation causing increase in their serum levels under hepatocellular injury or inflammation of the biliary tract cells resulting predominantly in an elevation of the alkaline phosphatase levels. On the other hand, elevation in ALP is usually indicates a cholestatic liver diseases.

Chronic administration of the four plants extracts for two weeks resulted in significant reduction in alkaline phosphatase levels. This shows that no possible cholestasis occurred at the dose levels tested since a rise in plasma ALP level is usually a characteristic finding in cholestatic liver diseases [50]. This was further confirmed by the fact that there were no significant changes in ALT and AST. Accordingly the plants extracts did not exhibit any signs of hepatotoxicity on chronic administration.

In this study, we were focusing on the liver injury that always accompanied by elevated levels of serum hepatic enzymes that are indicative of cellular leakage [55]. The hepatotoxic effects induced by CCl_4_ are related to its active metabolite trichloromethyl radical, ^•^CCl_3_, this is manifested by marked elevation in the serum liver enzymes namely AST, ALT, ALP [52]. Antioxidant enzymes, particularly, CAT, GSH-Px and SOD play a vital role in protecting cells against oxidative damage. Administration of CCl_4_ to the animals leads to induction of hepatic oxidative stress that is characterized by significant decrease in CAT, GSH-Px and SOD activities and increased TBARS content in liver tissue [52].

**Fig. 3:**
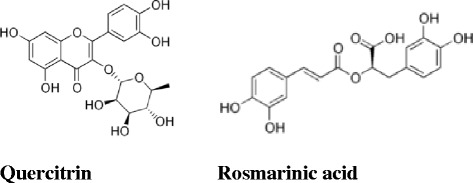
Chemical structures of quercitrin and rosmarinic acid

It was found that phenolic, especially polyphenolic, compounds such as flavonoids are very efficient scavengers of free radical**s** [56] because of their molecular structures, which include an aromatic ring with hydroxyl groups containing mobile hydrogen. Actually, it is known that the 3',4'-*ortho*-dihydroxy group in the B-ring and the 5-OH group in the A-ring with a 4-carbonyl group are required for the high antioxidant activity of flavonoids. In addition, the presence of the *o*-catechol group (3',4' -OH) in the flavonoid-B ring is a determinant for high antioxidant capacities in flavonoids (Fig. 3), [57]. The flavonoid content of *Fagonia indica* exceeded those of the other plants by 10 to 30 folds. The phenolic content of *Salsola imbricata* extract was previously reported to be 0.571 % [39]; while *Calotropis procera* extract showed high phenolic acids content. The frequently used DPPH assay was applied as a first *in vitro* approach to assess the free radical-scavenging activity of the plants extracts prepared in the different solvents. This evaluation revealed that the methanolic and ethanolic extracts of the four plants exhibited the highest DPPH radical scavenger potential with activity comparable to those of ascorbic acid, a well-known antioxidant. However, for safety and economic considerations, the ethanol (70 %) was selected for further biological study.

Dose selection for the subsequent biological study for the three plants (*Zygophyllum hamiense, Calotropis procera and Salsola imbricata)* was based on their LD_50_ values (less than 1/10 of LD_50_). Moreover, the major influencing factors to select smaller doses for *Fagonia indica* extract (5 and 10 mg/kg) was the extremely high contents of flavonoid and phenolic acid compared to the other plants extracts. Additionally, similar doses of *Fagonia indica* were tested previously for its analgesic effect [11].

The three plants, *Fagonia indica, Calotropis procera* and *Salsola imbricata* possessed high antioxidant and hepatoprotective activities. These effects may be attributed to the presence of high content of different groups of phenolic compounds including flavonoids aglycone and/or glycosides and phenolic acids especially quercetrin glycoside and rosmarinic acid (Fig. 3) that have been earlier reported to exhibit strong antioxidant and hepatoprotective effects [58, 59]. Moreover, hesperidin and its aglycone hesperitin, apigenin and cinnamic acid that exhibited strong antioxidant and hepatoprotective activities [60–64] were detected in all plants extracts except *Zygophyllum hamiense* of which rutin was the predominant flavonoid (10.71 %)*.* The presence of high rutin concentration in *Zygophyllum hamiense* extract explains its *in vitro* free radical scavenging activity and possibly it’s *in vivo* effect on AST level. Anyway, the plant could not be considered as hepatoprotective since ALT level, that is thought to be more specific for hepatic injury [54], was not significantly improved. Accordingly, the lack of other flavonoids especially quercetrin that presents in the other three plants explains the weak *in vivo* antioxidant and hepatoprotective activities of *Zygophyllum hamiense* against CCl_4_ intoxication.

Interestingly, it was found that although S*. imbricata* contains the lowest amount of phenolic contents, its efficacy in reducing ALT level in CCL_4_ treated mice still high. This can be explained by the presence of triterpenoid saponin. Two triterpenoidal saponin glycosides were isolated and identified from the *S. imbricata*, namely, salisomide and salisoflavan [38], and it is reported in literature that saponins interact with and increase permeability of the mucosal cells in the gut and enhance the absorption of various nutrient [65, 66]. Therefore, saponin enhanced the absorption of phenolic compounds. Additionally, saponins themselves possess antioxidant activity that contributes to efficacy of the phenolic compounds to protect against liver injury induced by CCL_4_ [67, 68].

Based on that, it was found that *F. indica, C. procera* and *S. imbricata* possess hepatoprotective effects by preventing the induction of oxidative stress and enhancing the hepatic antioxidant defences involving CAT, GSH-PX and SOD enzymes and were highly efficient in reducing the TBARS content in liver tissue. Accordingly, the three plants could be added to the growing list of medicinal plants. Further clinical studies are needed to evaluate their clinical significance.

## Conclusion

In conclusion, this study evidences the efficiency of the ethanolic extracts of *F. indica*, *C. procera* and *S. imbricata* in preventing the CCl_4_-induced hepatotoxicity in mice through scavenging of free radicals and by boosting the antioxidant capacity of the liver. The bioactive antioxidant principles detected in the extracts are probably responsible for this hepatoprotective effect. Therefore the parent plants could be considered as a potential source of safe protective from liver diseases or for reduction of undesirable hepatotoxic side effects of some drugs.
